# Structure and Properties of Barium Titanate Lead-Free Piezoceramic Manufactured by Binder Jetting Process

**DOI:** 10.3390/ma14164419

**Published:** 2021-08-06

**Authors:** Vadim Sufiiarov, Artem Kantyukov, Anatoliy Popovich, Anton Sotov

**Affiliations:** Institute of Mechanical Engineering, Materials, and Transport, Peter the Great St. Petersburg Polytechnic University, 195251 Saint Petersburg, Russia; kantyukov.artem@mail.ru (A.K.); director@immet.spbstu.ru (A.P.); SotovAnton@yandex.ru (A.S.)

**Keywords:** additive manufacturing, binder jetting, lead-free piezoceramic, barium titanate, sintering, piezoelectric properties

## Abstract

This article presents the results of manufacturing samples from barium titanate (BaTiO_3_) lead-free piezoceramics by using the binder jetting additive manufacturing process. An investigation of the manufacturing process steps for two initial powders with different particle size distributions was carried. The influence of the sintering and the particle size distribution of the starting materials on grain size and functional properties was evaluated. Samples from fine unimodal powder compared to coarse multimodal one have 3–4% higher relative density values, as well as a piezoelectric coefficient of 1.55 times higher values (d_33_ = 183 pC/N and 118 pC/N correspondingly). The influence of binder saturation on sintering modes was demonstrated. Binder jetting with 100% saturation for both powders enables printing samples without delamination and cracking. Sintering at 1400 °C with a dwell time of 6 h forms the highest density samples. The microstructure of sintered samples was characterized with scanning electron microscopy. The possibility of manufacturing parts from functional ceramics using additive manufacturing was demonstrated.

## 1. Introduction

Functional ceramics are a class of materials that exhibit special properties in addition to those already inherent in ceramics, such as chemical and thermal stability. Functional ceramics typically exhibit one or more unique properties: biological, electrical, magnetic, or chemical. [1]. Due to this, they are used in engine production, aviation, and space industries [2]. The most promising types of functional ceramics include piezoceramics [3]. Piezoelectric ceramics are used to make sensor devices, energy harvesters, and actuators [4,5,6]. Piezoelectric materials are of particular interest as pressure and temperature sensors in high-frequency environments [7,8]. Piezoelectric ceramics have generated particular interest in the power industry because they can withstand the harsh environmental conditions present in energy conversion systems [9]. However, despite their advantages as sensitive devices, piezoceramics also have the same internal disadvantages that are observed in most ceramic materials: they are difficult to process [10], and their fragility causes low fracture resistance [11]. Therefore, the manufacture of non-standard complex geometries from ceramic materials can be practically impossible using conventional manufacturing methods. The proposed method for circumventing this problem is the manufacture of complex ceramic parts using additive manufacturing (AM) [12]. AM has such advantages as the absence of expensive tools, easy scalability of the process, the ability to implement parts of complex shapes, a high degree of material utilization and minimum production time [13]. One of the most relevant materials for ceramic additive manufacturing is a piezoelectric material since it generates an electric charge when deformed or, conversely, deforms when an electric potential is applied. The use of AM for the manufacture of piezoelectric materials will expand the scope of their application, expanding the possibilities of forming multilayer, as well as complex geometries of structures. Therefore, the possibility of integrating piezoelectric materials during the manufacturing process itself would lead to the creation of multifunctional structures within a single processing process. With great freedom in the achievable geometries of piezoelectric elements, the prospect opens for a significant improvement in the performance of many devices based on piezoelectric and ferroelectric properties [14,15].

Some previous investigations of additive manufacturing lead-based piezoceramics have been made using direct writing/FDM [16,17], stereolithography-based processes [18,19], and ink-jetting [20]. Due to the toxicity of lead compounds, the development of new piezomaterials and technologies is moving towards lead-free piezomaterials. Barium titanate (BaTiO_3_) is one of the most widely used lead-free piezoceramic materials, which became widespread due to its high dielectric and piezoelectric properties [21]. The most promising methods of 3D printing BaTiO_3_ are direct writing (DW) [22,23,24,25], vat photopolymerization (VP) [26,27,28,29,30], and binder jetting (BJ) [31,32,33,34]. Samples printed using DW have the best piezoelectric coefficient values (d_33_ = 200 pC/N [22]) and a density (6.01 g/cm^3^ [23]) close to the theoretical density limit of BaTiO_3_ (6.02 g/cm^3^). However, the quality of the surface printed layer is rather rough, which may be a limitation for using this technology. It is also worth noting that difficulties arise when using printing nozzles with a diameter less than 500 microns—the nozzle can clog with ceramic powder particles, and this reduces the accuracy of printing parts [23]. Samples printed using VP also have high piezoelectric coefficient values (d_33_ = 165 pC/N [27] and high material density (5.64 g/cm^3^ [26]). However, there are next limitations when using this 3D printing technology [29]: (i) using 3D printer with a layer spreading system for viscous slurry with a high solid loading of ceramics in the photopolymer resin; (ii) the high refractive index of UV light for BaTiO_3_ that limits the curing depth; (iii) a long time of debinding process that directly affects the final result of the subsequent sintering. Samples printed using BJ have low piezoelectric coefficient values (d_33_ = 74.1 pC/N [31], d_33_ = 112 pC/N [32] and have a low density (3.93 g/cm^3^ [31], 2.21 g/cm^3^ [32]). However, this technology ensures the high quality of printed parts and excludes difficulties of the debinding process.

The BJ additive process is a method where a nozzle print-head jets a liquid binder on a powder layer in places that correspond to the cross-section of the computer model of a part. The result of BJ printing is a green model with low mechanical properties and high porosity. The green model needs further curing, debinding, and sintering. As a result, the characteristics of parts made of the polymer [35], metal [36,37,38] and ceramics [39] printed on a 3D printer largely depend on manufacturing and postprocessing parameters. Consequently, the behavior of functional ceramics made with additive technologies must be further studied to expand the capabilities of this new technique.

In this paper, BaTiO_3_ lead-free piezoceramic was used to study the additive manufacturing of piezoelements by using the BJ process. The influence of the manufacturing process on the properties of the material was characterized and discussed, and the dielectric and piezoelectric properties of the manufactured samples were measured.

## 2. Materials and Methods

### 2.1. Materials

**Figure 1:**
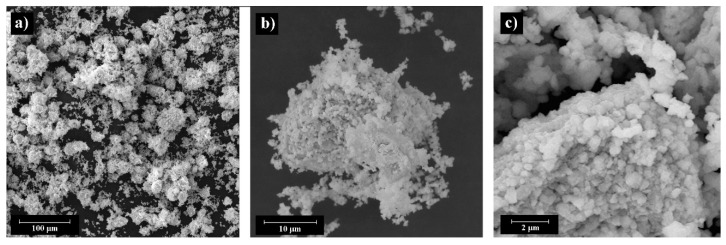
SEM images of C-BaTiO_3_ powder at different magnifications: general view (**a**), agglomerate (**b**), powder particles (**c**).

**Figure 2:**
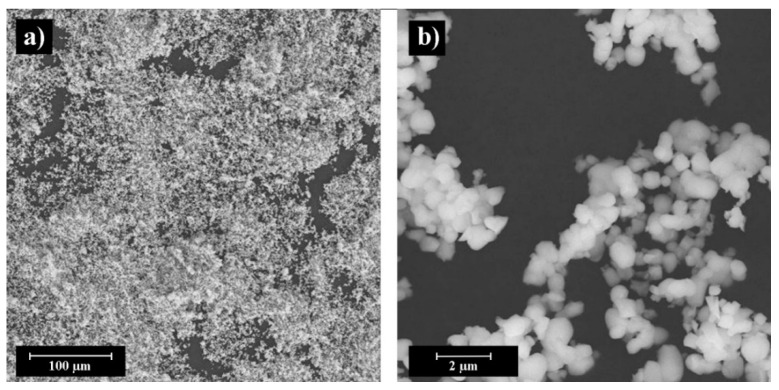
SEM images of F-BaTiO_3_ powder at different magnifications: general view (**a**), powder particles (**b**).

**Figure 3:**
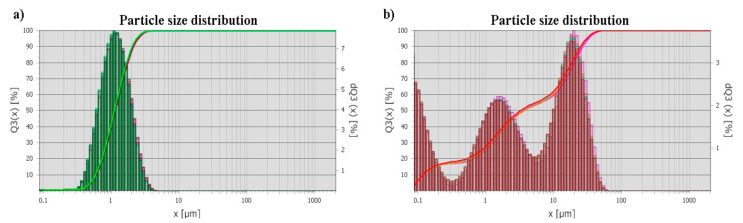
Particle size distribution of F-BaTiO_3_ (**a**) and C-BaTiO_3_ (**b**).

Two types of BaTiO_3_ powder were used for printing by BJ: (i) micron powder with multimodal particle size distribution (PSD) (C-BaTiO_3_, ZAO NPF Luminofor, Stavropol, Russia) D_10_—0.1 µm, D_50_—3.4 µm, D_90_—25.4 µm, and (ii) submicron powder with unimodal PSD (F-BaTiO_3_, Acros Organics, Geel, Belgium) D_10_—0.6 µm, D_50_—1.1 µm, D_90_—2.1 µm. Figure 1 and Figure 2 shows images of as-received powders. The C-BaTiO_3_ powder has particle sizes of about 1 μm, forms agglomerates up to 25 μm (Figure 1). The F-BaTiO_3_, powder has particle sizes about 1 μm (Figure 2). Figure 3 shows the particle size distributions which demonstrates that the C-BaTiO_3_ powder consists of agglomerates and reveals three peaks and contains small particles. The unimodal powder is much more homogeneous, while in the case of the multimodal powder one can observe the agglomeration of small particles into larger clusters, which correspond to the third peak in the PSD with a medium size at about 20 microns.

### 2.2. Fabrication

For the experiment green models of cubic shape with dimensions of 10 × 10 × 10 mm^3^ were printed to study subsequent debinding and sintering processes. Also, the two types of cylindrical green models (diameter 15 mm and 10 mm, height 10 mm and 1 mm respectively) were printed to investigate the electromechanical properties.

The piezoceramic samples were manufactured on the ExOne Innovent system (The ExOne Company, North Huntingdon, PA, USA). This system relates to the BJ additive manufacturing process. The original ExOne BS004 solvent binder and CL001 cleaner were used for the printing of the functional ceramic components.

**Figure 4:**
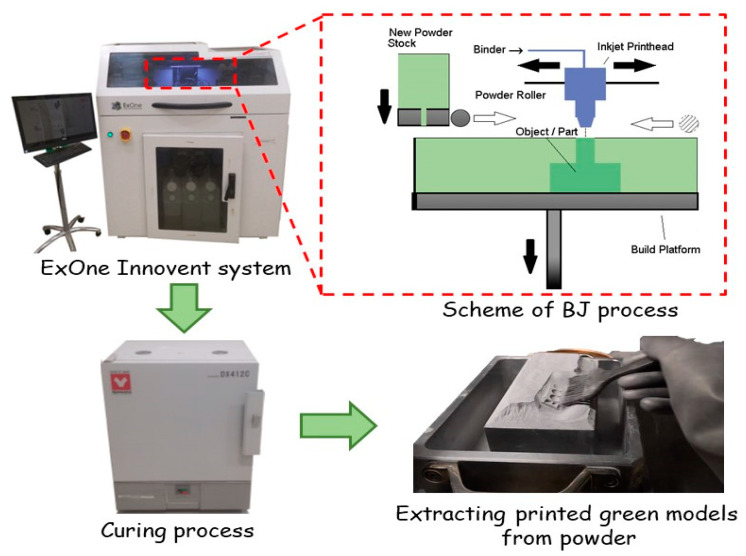
Process flow for BJ additive manufacturing.

The BJ process can be divided into several stages, a schematic image of which is shown in Figure 4:

1. A thin layer of powder material is formed on the platform using a roller;
2. A liquid binder is selectively sprayed to the powder layer using a print head, in accordance with the cross-section of the computer model;
3. Then the platform is lowered to a given thickness of one layer;
4. The powder layer is dried and heated using an infrared heater;
5. From the hopper, using an oscillator, the powder is fed to the surface of the platform and a new layer of powder is applied;
6. Then the layer is leveled using a rotating roller;
7. Processes 1–6 are repeated until a full-size green model is made.

Printed parts are considered “green” and are not suitable for end-use. Thus, these green models require further post-processing, such as sintering or infiltration, to achieve the desired mechanical and functional properties.

After the 3D printing, the platform (together with the green models in the powder surround) is placed in the thermal furnace (Yamato DX412C, Yamato Scientific, Santa Clara, CA, USA) at 180 °C for 3 h for curing.

After curing, the green models have sufficient strength to remove excess and loose powder. For green models of a simple shape, removal was done with a brush; for complex shapes removal was done using compressed air.

### 2.3. Thermal Post-Treatment

**Figure 5:**
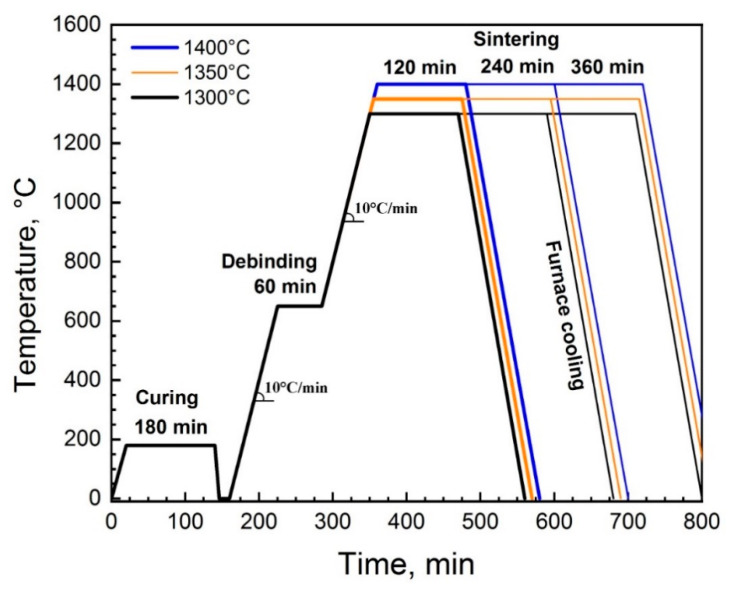
The thermal post-treatment profile.

Before thermal post-treatment, the green models were placed in alumina crucibles with lids. The debinding process was performed in a muffle furnace (KJ-1700X, Zhengzhou Kejia Furnace Co., Ltd., Zhengzhou, China) at a temperature of 650 °C with a dwell time of 60 min. After debinding, the samples were sintered in a muffle furnace at 1300, 1350, and 1400 °C with a dwell times of 2, 4, and 6 h under an air atmosphere. The thermal post-treatment profiles are illustrated in Figure 5.

### 2.4. Characterization

The particle size distribution of the powders was determined by laser diffraction Analysette 22 NanoTec plus (Fritsch, Idar-Oberstein, Germany) with a total measurement range of 0.01–2000 μm.

TGA analysis of BS004 solvent binder was performed using a thermogravimetric analyzer (Q5000, TA Instruments, New Castle, DE, USA). The heating was carried out in an airflow of 30 mL/min in the temperature range 30–700 °C at a rate of 10 °C/min. The binder was placed in a platinum crucible, after which it was heated from room temperature up to 700 °C in air.

The structure of the samples after sintering was studied using a Leica DMI5000 optical microscope (Leica, Wetzlar, Germany) and a Tescan Mira3 LMU scanning electron microscope (SEM) operating at magnifications from 4× to 10^6^× with an accelerating voltage from 200 V to 30 kV. The chemical composition was measured using an energy-dispersion accessory into the SEM.

Optical and SEM-images of sintered samples from C-BaTiO_3_ and F-BaTiO_3_ were examined using the ImageJ Software. v.1.52a (Bethesda, MD, USA) The grain sizes were analyzed for various temperature and time sintering conditions.

The density of the sintered samples was measured by the Archimedes method; the calculation of relative density was made in accordance with the theoretical density of BaTiO_3_ (6.02 g/cm^3^).

The phase composition was analyzed using a Bruker D8 Advance X-ray (Bruker corp., Billerica, MA, USA) diffractometer (XRD) using CuKa radiation (l = 1.5418 Å) without monochromator.

All samples for the electrical performance test were coated with silver electrodes (paste PP-17, Delta, Zelenograd, Russia) at 700 °C for 30 min. The samples were poled in air, at Tc + 20 °C (Tc-Curie temperature 120–130 °C for BaTiO_3_). Then, an electric field of 0.6 kV/mm for 30 min was applied to samples, followed by cooling to room temperature. Dielectric constant έ, the loss tangent tgδ, electromechanical coupling coefficient k_p_, and piezoelectric coefficient d_33_ were measured and calculated. Dielectric properties were measured on cylindrical samples with a diameter of 10 mm and a height of 1 mm. The capacity of the sample and the loss tangent were measured with an E7-28 immittance analyzer at 1 kHz frequency at 0.5 V effective voltage. The piezoelectric coefficient d_33_ was determined on polarized cylindrical samples using the APC YE2730A setup by a quasi-static method. The values of the electromechanical coupling coefficient were calculated by the following equation:

(1) $$k_{p}=\sqrt{\frac{\delta_{p}}{a_{p}+b_{p}\cdot \delta_{p}},}$$

where, a_p_, b_p_ are the coefficients determined of Planar Poisson’s Ratio, δ_p_ is the relative resonance gap. The Planar Poisson’s ratio value was determined by the frequencies ratio of the third and first (main) overtones of the planar vibration mode on piezoelectric elements in the form of a disk.

## 3. Results and Discussion

### 3.1. Investigation of Debinding Process

**Figure 6:**
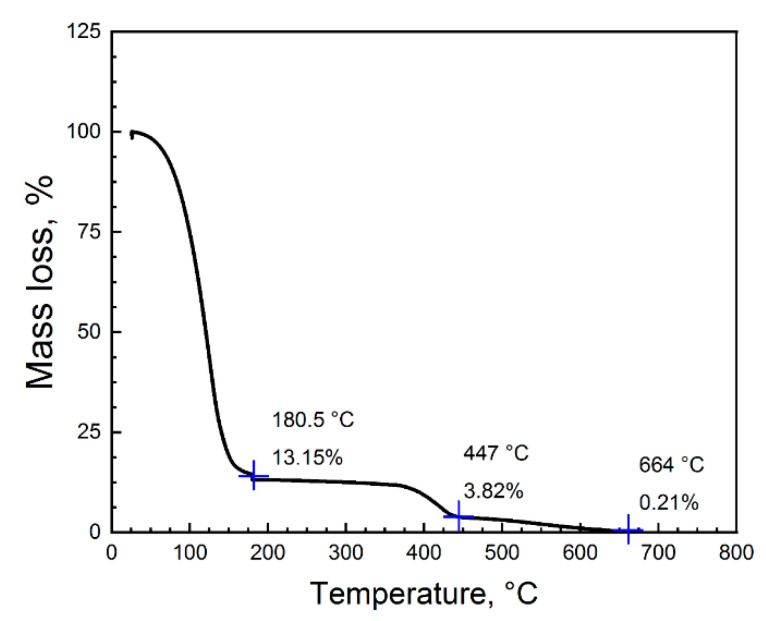
TGA analysis of the binder.

The TGA curve showed that when heated to 180.5 °C a sharp mass decrease by 86.82% was observed (Figure 6). This is due to the evaporation of two components: ethylene glycol monobutyl ether (EGBE), isopropanol (IPA), and the polymerization of ethylene glycol to polyethylene glycol. The boiling temperatures of evaporating components are much lower at 171 °C and 80.4 °C, respectively.

A further mass decrease occurred at a temperature range of 380–450 °C. As a result, the remaining mass of the binder was 0.82% of the initial one. Increasing the temperature leads to a linear decreasing of mass; the binder was almost completely thermally decomposed at 664 °C and the residue was 0.21% of the initial mass. Thus, mass loss of the binder is observed in two stages: the first stage—mass decreases on 86.85%, this stage ends at a temperature of 180.5 °C. The second stage is the temperature range from 180 to 664 °C. Here, from 180 to 447 °C, no significant mass loss occurs. From 447 to 664 °C, the mass loss is up to 0.21% of the original mass.

The first stage is associated with the transition of ethylene glycol to polyethylene glycol during curing, the second stage is debinding by the burn out of the residue components of the binder [40].

### 3.2. Binder Jetting Process

For the BJ process, the recoating speed (28 mm/s and 65 mm/s for C-BaTiO_3_ and F-BaTiO_3_ respectively) and the frequency of the oscillator (5000 rpm and 4400 rpm for C-BaTiO_3_ and F-BaTiO_3_ respectively) were previously optimized to apply a sufficient amount of material to form a smooth thin powder layer. Considering this, the layer thickness for C-BaTiO_3_ and F-BaTiO_3_ powder was 100 µm and 35 µm, respectively. The drying time and temperature were also optimized to achieve a uniform layer without cracking and without smearing. The main BJ parameters are shown in Table 1.

Further, the saturation parameter was investigated. Binder saturation is a computed value used to quantify how much binder is dispensed into each unit volume of powder material. Improper saturation of the binder can cause an inhomogeneous layer of powder as well as inaccurate dimensions of printed parts. The theoretical binder saturation (%) was estimated using the following equation:

(2) $$S=\frac{1000\times V}{\left(1-\left(\frac{\mathrm{PR}}{100}\right)\right)\times X\times Y\times Z},$$

where V is the volume of binder per drop (pL), PR is the packing rate (%), X and Y are the spacing between binder droplets (μm), and Z is the layer thickness (μm). To obtain the green part with sufficient mechanical strength and surface quality, optimizing the saturation level is critical.

The saturation for C-BaTiO_3_ powder varied from 40 to 140% with a step of 20%. For F-BaTiO_3_ powder, the saturation varied from 50 to 200% with a step of 50%. When printing the C-BaTiO_3_ and F-BaTiO_3_ samples, no defects were observed on the surface of the powder layer. The powder layer was applied uniformly, the particles did not stick to the roller. After curing of C-BaTiO_3_ samples printed at 40% saturation, the green model delaminated. At 60% and 80% saturation, the deviation from the computer model size amounted to 0.37 mm along the X and Y-axes and more than 0.1 mm along the Z-axis. For 100% saturation, the C-BaTiO_3_ samples had clear boundaries and the deviation from the computer model size was about 0.2 mm along the X and Y-axes, and less than 0.05 mm along the Z-axis. At 120% and 140% saturation, the geometry of the green models changed significantly and appeared to be barrel-shaped.

After curing of F-BaTiO_3_ samples printed at 50% saturation, the green model delaminated since there was not enough binder to bond the layers together. For 100% saturation, the F-BaTiO_3_ samples had clear boundaries and the deviation from the computer model size was about 0.2 mm along the X and Y-axes, and less than 0.02 mm along the Z-axis. At 150% and 200% saturation, the deviation from the computer model size amounted to 0.36 mm and 0.38 mm along the X and Y-axes and more than 0.21 mm and 0.25 along the Z-axis, respectively.

### 3.3. Investigation of Sintering Process, Shrinkage, Microstructure, Porosity

For investigation of sintering process BaTiO_3_ samples, the following samples were selected: C-BaTiO_3_ samples printed at 60, 80, and 100% saturation; F-BaTiO_3_ samples printed at 100, 150, and 200% saturation.

**Figure 7:**
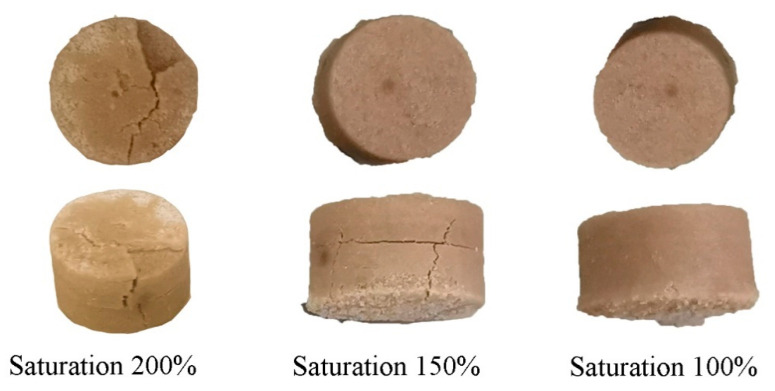
Images of F-BaTiO_3_ samples printed by BJ with different saturation after sintering. Scale: each sample has a diameter of 15 mm and a height of 10 mm. Heating rate of 10 °C/min to 1400 °C with a dwell time of 4 h.

To understand the influence of saturation level test-sintering was carried out at 1400 °C with a dwell time of 4 h. Samples of C-BaTiO_3_ printed with 60 and 80% saturation after test-sintering delaminated due to the weak contact between the layers. Also, F-BaTiO_3_ samples cracked at 150 and 200% saturation, which is due to the high content of the binder. Seemingly, due to the high content of the binder in samples, during debinding and subsequent sintering, the formation of a large amount of gas occurred, leading to the appearance of cracks. However, the C-BaTiO_3_ and F-BaTiO_3_ samples printed at 100% saturation after test-sintering were free from defects. Figure 7 shows an image of F-BaTiO_3_ samples obtained at different saturations after test-sintering. As a result, samples with 100% saturation for both types of powder were selected for further investigation of the sintering process.

Subsequently, these samples were subjected to sintering in the temperature range of 1300–1400 °C for 2–6 h. Sintering experiments at temperature 1500 °C led to the melting of BaTiO_3_ and the destruction of the samples. Initially, the study of the sintering process was carried out for C-BaTiO_3_ samples at various temperatures of 1300, 1350, and 1400 °C. The best value for the density of the material was achieved at 1400 °C. Considering that the particle size of the C-BaTiO_3_ powder is close to the particle size of the F-BaTiO_3_ powder (but different agglomerates sizes), a further investigation of sintering for the F-BaTiO_3_ samples was carried out at a temperature of 1400 °C with dwell times of 2, 4, and 6 h.

**Figure 8:**
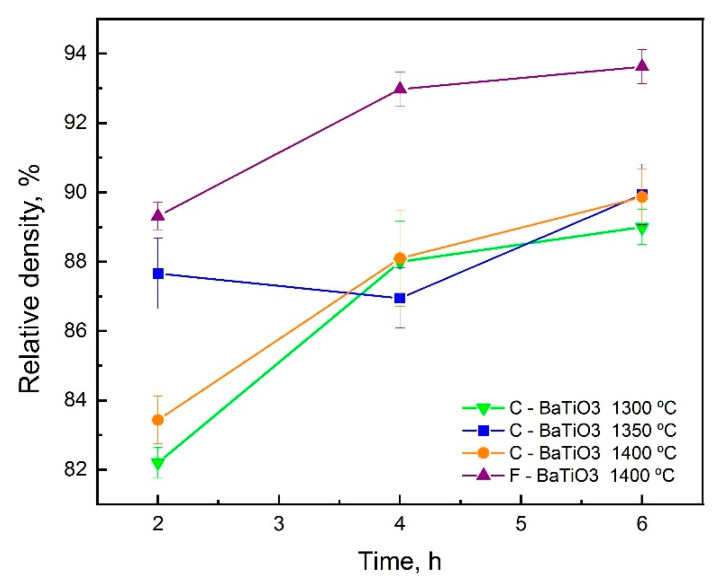
Dependence of the density of samples on temperature and dwell time of sintering.

Figure 8 shows graphs of the dependence of sintered samples density on dwell time. The density of the samples increases with an increasing dwell time. The density of F-BaTiO_3_ samples is higher compared to C-BaTiO_3_ samples. The density of C-BaTiO_3_ samples is lower, but the printing speed is higher due to layer thickness difference. During the printing of cylindrical samples from F-BaTiO_3_ with 15 mm diameter and 10 mm height, the printing time was 7 h, which is 4 h longer than the printing time for similar size samples from C-BaTiO_3_ powder.

**Figure 9:**
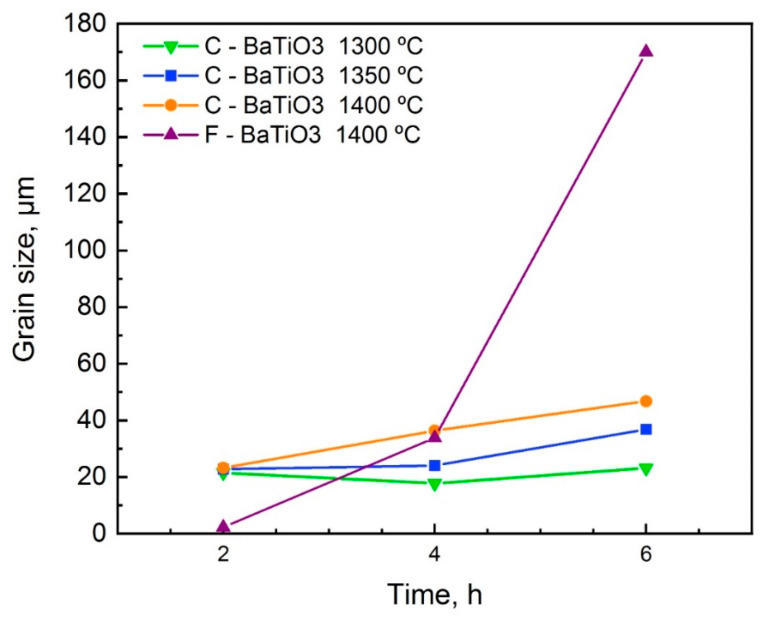
Grain size of BaTiO_3_ samples dependence on temperature and dwell time of sintering.

Increasing the temperature and dwell time of sintering leads to grain enlargement. The graphs in Figure 9 show that the grain size of samples from the unimodal powder is more sensitive to changes in temperature and dwell time compared to samples made from multimodal powder. This feature allows adjusting the functional properties of the material in a wider range.

As a result of the sintering of samples from C-BaTiO_3_, the shrinkage along the XY direction was 20–25%. The Z-axis shrinkage varied from 24.1% to 24.4%. The measured linear shrinkage of samples from F-BaTiO_3_ along the XY direction was 24–27%. The Z-axis shrinkage was 25–26%.

**Figure 10:**
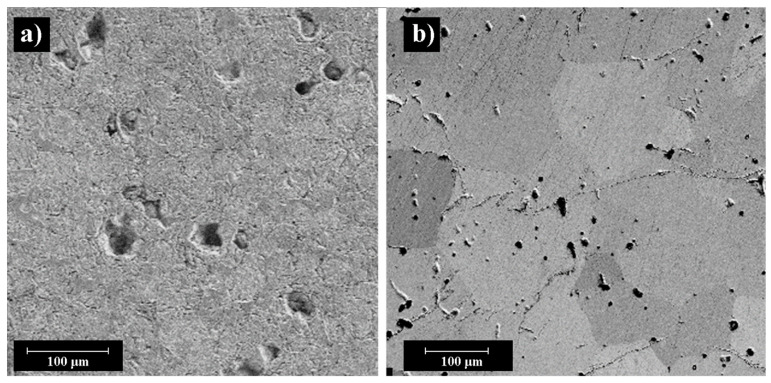
SEM images of sintered samples microstructures at 1400 °C, a dwell time of 6 h from C-BaTiO_3_ (**a**) and F-BaTiO_3_ (**b**) powders.

The microstructure of the sintered BaTiO_3_ samples is shown in Figure 10. The structure is a rounded grain formed as a result of sintering the powder material. Some sintered samples have round-shaped pores, these defects may be associated with binder removal since at this stage there is active gas formation, and perhaps a consequence of the non-optimal sintering mode as well.

**Figure 11:**
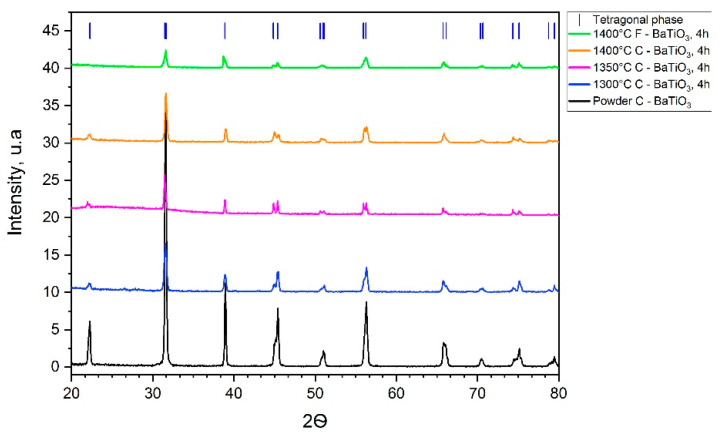
XRD of a sample sintered at different temperatures.

According to EDS measurements, the chemical composition of samples was 59.2% of Ba, 18.8% of Ti, and 22% of O (weight %) which corresponds with BaTiO_3_ formulation. Figure 11 shows the diffraction patterns of the C-BaTiO_3_ samples. X-ray diffraction analysis showed that all samples are composed of the tetragonal crystal lattice P4mm of BaTiO_3_, as evidenced by bifurcated peaks (compare to cubic lattice Pm-3m).

**Figure 12:**
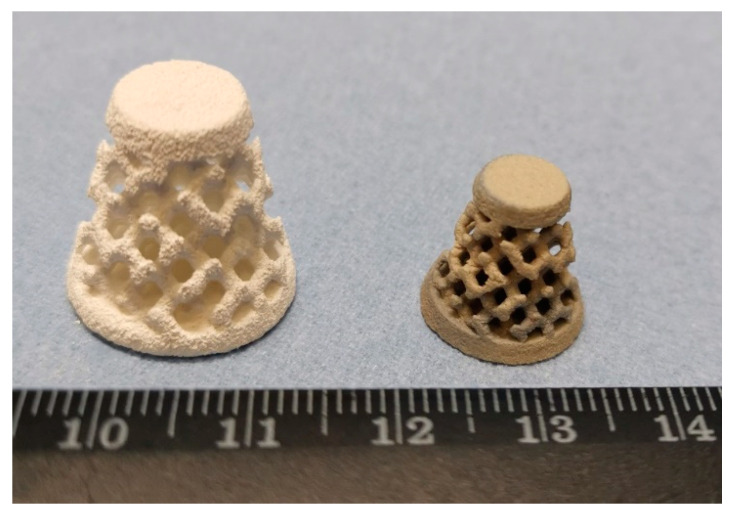
Image of samples with lattice structures printing by BJ from F-BaTiO_3_ powder before (**left**) and after sintering (**right**).

To demonstrate the applicability of the developed modes of the BJ process and thermal post-treatment for manufacturing parts with complex geometries, test samples with lattice structures were made from F-BaTiO_3_ powder (Figure 12).

### 3.4. Investigation of Functional Properties

The investigation of the functional piezoelectric properties was carried out for C-BaTiO_3_ samples (a temperature of 1400 °C and a dwell time of 6 h) and F-BaTiO_3_ (a temperature of 1400 °C and a dwell time of 4 h). These samples were selected considering the highest density and grain size up to 50 microns. This grain size is due to the fact that, for BaTiO_3_-based piezoceramic, the high functional properties arise with a grain size of 10 to 50 μm [41]. Table 2 shows the test results of the functional properties of sintered samples manufactured from multimodal and unimodal BaTiO_3_ powders. Samples printed from C-BaTiO_3_ powder are inferior in dielectric constant, electromechanical coupling coefficient, and piezoelectric coefficient to samples printed from F-BaTiO_3_. This can be explained by the non-optimal mode of debinding and sintering, the presence of large pores, and as a result, a decrease of the active phase volume of the sample.

Appreciating the main parameter piezoelectric coefficient d_33_, it can be noted that using the BJ process allows achieving 72.4% of the piezoelectric coefficient compared to the value obtained by traditional manufacturing technology with multimodal PSD powder and 79.6% of the d_33_ values obtained with unimodal PSD powder. Pressing and sintering were used as the traditional technology, and a solution of polyvinyl alcohol was used as a binder. Sintering was carried out at a temperature of 1350 °C, heating rate 100 °C/h, a dwell time of 3 h.

According to the results of studies published in [32], the functional characteristics of AM piezoceramics depend on the direction of measurement. The functional properties along the Z-axis are about 20% smaller in comparison with the XY direction. In the current study, the properties were measured only along the Z-axis, but the achieved values of piezoelectric coefficient d_33_ = 183 pC/N and dielectric constant έ = 811 exceed the values obtained by the authors [32] parallel (d_33_ = 113 pC/N, έ = 581.6) and perpendicular to the printing orientation (d_33_ = 152.7 pC/N, έ = 698). These differences seem to be related to the raw material and the corresponding difference in technological parameters of BJ and subsequent thermal post-treatment.

The presented results demonstrate that the use of a unimodal PSD powder of lead-free piezoceramics barium titanate allows achieving higher piezoelectric properties, and the use of binder jetting technology allows the creation of objects with complex geometry, which has potential in the manufacture of ultrasonic products used in medicine, aviation, marine industry, sensors for monitoring welded joints, pressure sensors in pipelines, etc.

Future research areas that allow for improving piezoelectric properties include the use of new lead-free piezoelectric materials with increased characteristics (such as KNN, BZT-BCT, etc.), as well as the creation of functional gradient systems and the use of multimaterial 3D printing.

## 4. Conclusions

The paper presents the results of the additive manufacturing of piezoelectric elements using the binder jetting process. Two powders with different particle size distributions were used as raw materials. Binder jetting with 100% saturation for C-BaTiO_3_ and for F-BaTiO_3_ allows printing samples without delamination and cracking. Sintering at 1400 °C with a dwell time of 6 h forms the highest density samples. It was determined that samples from the unimodal powder are more sensitive to increasing grain size during sintering. The measured dielectric and piezoelectric properties of the samples also demonstrated that samples from unimodal powder F-BaTiO_3_ have higher values. The results of the functional piezoelectric properties obtained by binder jetting with C-BaTiO_3_ are d_33_ = 118 pC/N, έ = 750, and with F-BaTiO_3_: d_33_ = 183 pC/N, έ = 811.

The future possibilities of improving functional characteristics of samples manufactured with BJ are increasing speed, optimizing sintering modes, and using new lead-free piezoelectric materials with improved functional characteristics.

## Figures and Tables

**Table 1:** The main BJ parameters for BaTiO_3_ lead-free piezoceramic powder.

Process Parameter	C-BaTiO_3_ Powder	F-BaTiO_3_ Powder
Recoating speed	28 mm/s	65 mm/s
Frequency of the oscillator	5000 rpm	4400 rpm
Layer thickness	100 µm	35 µm
Drying time	25 s	20 s
Drying temperature	25 °C	33 °C
Roller movement speed	1 mm/s	1 mm/s

**Table 2:** Piezoelectric properties at 1 kHz of BaTiO_3_ samples printed by BJ process.

Technology/Powder Type	έ	tgδ, %	k_p_	d_33_, pC/N
Binder Jetting/C-BaTiO_3_	750	5.53	0.15	118
Traditional technology/C-BaTiO_3_	1872	7.9	0.22	163
Binder Jetting/F-BaTiO_3_	811	11.59	0.19	183
Traditional technology/F-BaTiO_3_	2367	1.7	0.36	230

## Data Availability

The data presented in this study are available on request from the corresponding author.
